# Electronic Structure and Spectral Properties of Radical Heterocyclocarbon C_12_N Isomers

**DOI:** 10.1021/acs.jpclett.6c01817

**Published:** 2026-08-25

**Authors:** Yuri Tanuma, Ihor Sahalianov, Rinat Nasibullin, Glib Baryshnikov

**Affiliations:** a Laboratory of Organic Electronics, Department of Science and Technology, 4566 Linköping University, SE-60174 Norrköping, Sweden; b Wallenberg Initiative Materials Science for Sustainability, Department of Science and Technology, 4566 Linköping University, SE-60174 Norrköping, Sweden

## Abstract

Cyclo­[*n*]­carbons are reactive ring-shaped carbon allotropes that have been theoretically and experimentally studied for various numbers of carbon atoms in the ring (*n*). Recently, the first heterocyclocarbon, C_12_N, was experimentally synthesized and its molecular structure confirmed using scanning tunneling microscopy by Sun et al., revealing a likely *C*_2*v*_-symmetric ring. In this paper, we report on further theoretical investigation on the C_12_N molecule and its isomers, which have different bond length alternation (BLA): cumulene-like structure with smaller BLA and polyyne-like one with larger BLA. Calculations confirm that the cumulene-like C_12_N structure is slightly more stable. Calculated BLA, bond angle alternation, isochemical shielding surface, magnetically induced ring current, and *g*-factor consistently indicate the aromaticity of the cumulene-like C_12_N and antiaromaticity/less-aromaticity of the polyyne-like C_12_N. In addition, calculated electron paramagnetic resonance and infrared and Raman spectroscopic data are reported for both isomers as potential features for experimental identification in the future.

## Graphical abstract

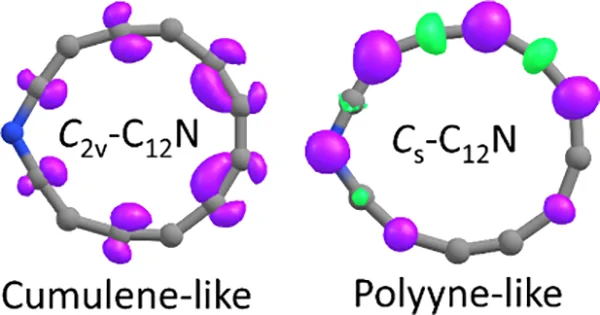

Since the first successful synthesis of cyclo[18]carbon in 2019,^1^ which enabled experimental analysis of highly reactive cyclo­[*n*]­carbon molecules, the structures and aromaticity of the cyclo­[*n*]carbon family have been intensively studied. To date, cyclo­[*n*]­carbons with *n* = 6,^2^ 10,^3^ 12,^4^ 13,^5^ 14,^3^ 16,^6^ 18,^1,7^ 20,^4,8^ 26,^5^ and 30 ^8^ have been experimentally synthesized. These molecules are typically formed via voltage pulses applied through a scanning tunneling microscopy (STM) tip to precursor molecules adsorbed on a NaCl surface.^1,5^ The pulses trigger the retro-Bergman ring-opening reaction, cleaving fragments from the precursor and resulting in the formation of cyclo­[*n*]carbon rings. Yield depends on precursor molecules.^7^

Smaller carbon rings are unstable due to the strong curvature, but larger ones are also difficult to keep the ring shape as the ring twists and forms bonds with itself. For overcoming such a weakness, supramolecular chemistry has been one of the effective approaches. Recently, Gao et al. successfully synthesized stable cyclo[48]­carbon-catenane complexes in solution by threading the C_48_ ring into 3 catenane rings, which sterically protect the cyclo[48]carbon ring structure from cross-linking.^9^ This realized spectroscopic characterization of cyclo[48]­carbons incorporated with the catenane rings; thus it is attracting further scientific attention to cyclo­[*n*]­carbons.

For this rapidly developing topic, theoretical studies are also intensively carried out. Besides wide range of even and odd “*n*” for cyclo­[*n*]­carbons, metal- ^10,11^ and boron-^11^ incorporated cyclocarbons have been investigated. Such modifications would adjust magnetic and electronic properties and molecular stability. Establishing stabilization methods for cyclo­[*n*]­carbons is of great importance for experimental analysis and practical application.

Cyclo­[*n*]carbon rings form either cumulenic structures with consecutive double bonds or polyynic structures with alternating single and triple bonds. Theoretical studies suggest that for smaller rings (*n* < 16), cumulenic forms are energetically favored.^12,13^ Nevertheless, due to their high reactivity, further detailed experimental characterization of cyclo­[*n*]­carbons beyond their molecular structure still remains challenging.

These carbon rings are of great interest not only for their unique structures and electronic properties but also from an astrochemical point of view. Polycyclic aromatic hydrocarbons (PAHs) have been detected in the interstellar medium (ISM),^14^ and over 300 molecules have been spectroscopically identified. One of the most notable assignments is the C_60_ fullerene.^15,16^ Regarding the formation of C_60_ in space, according to recent studies, the top-down process is a leading theory based on its observed abundance and experimental efficiency.^17^ However, polyynic structures may still play a crucial role in the growth of PAHs into C_60_, as polyyne chains are seen in graphite at high temperature,^18,19^ shrinkage simulation of larger PAH,^20^ and simulation of clustering C_2_ fragments.^20^ Damaging ring and planar polyaromatics would also create polyyne chain in the structure.^21,22^ Furthermore, recent theoretical studies suggest that fragmentation of several PAHs converges into ionised cyclo­[*n*]carbon rings (*n* = 11–15).^23,24^ From these perspectives, cyclo­[*n*]­carbons may be a missing piece of puzzle of the carbon cycle within the ISM.

Sun et al. recently reported the surface synthesis of a heterocyclocarbon ring, C_12_N, which is the first experimental observation of heterocyclo­[*n*]carbon ring.^25^ The experimental synthesis was via the retro-Bergman ring-opening reaction on a precursor by the STM voltage-pulse method.^25^ Owing to its highly curved ring structure with an unpaired electron, the C_12_N molecule cannot be experimentally analyzed outside the STM environment. However, the STM observation and theoretical investigation revealed that the experimentally obtained C_12_N takes a ring structure with localized distortion at the nitrogen site.^25^ The calculated isochemical shielding surface (ICSS_*zz*_) plot shows the presence of diatropic ring current, indicating that the system is aromatic.^25^

In theoretical studies on such aromatic molecules, resonance structures are usually obtained by density functional theory (DFT) calculations. However, in this study, we found a new C_12_N isomer with polyyne-like structure, which exhibits a different electronic structure and molecular orbital (MO) shapes from the previously obtained cumulene-like structure. Note that the notations “cumulene-like” and “polyyne-like” are defined by their bond length alternation (BLA), i.e., structure with smaller BLA is cumulene-like, and one with larger BLA is polyyne-like, in this paper. C_12_N is particularly an interesting molecule for fundamental studies because of its odd-membered ring and the presence of a heteroatom, which results in the radical character. Therefore, we characterized their electron paramagnetic resonance (EPR) properties in addition to the basic molecular information and parameters involved with aromaticity, since the EPR is a powerful technique for radical analysis, capable of contributing to identification of molecular structures. The aim of this study is to provide basic insights into the aromaticity of different C_12_N conformers, thereby supporting future efforts to stabilize related cyclo­[*n*]carbon molecules by tuning their aromaticity. This work promisingly contributes to further exploration on a new class of carbon allotropes: 0D carbon rings without hydrogen termination.

Density functional theory simulations were carried out with B3LYP,^26,27^ BHandHLYP,^28^ BMK,^29^ M062X,^30^ PBE,^31^ and ωB97XD^32^ exchange–correlation functionals and 6-311++G** ^33–37^ basis set by using the Gaussian software (version 16.C.01).^38^ Isochemical shielding surfaces (ICSS_*zz*_) were simulated by using the NMR shielding tensors calculated by gauge-independent atomic orbital GIAO approach^39^ and then visualized using the Multiwfn 3.8 software.^40^ Anisotropy of induced current density (AICD) plots were calculated by a method developed by Herges et al.^41^ After the geometric relaxation, frequency calculation was performed and no imaginary frequency was confirmed. The ChemCraft^42^ software was used for visualization.

For the *ab initio* calculation part, SA-CASSCF­(11,11)^43–45^ was used both to optimize the molecular geometries and to analyze the wave function character of the low-lying electronic states at different geometries. Calculations were performed using ORCA 6.0.1 ^46^ with the def2-TZVP basis set,^33–35,46^ averaging over four doublet states (D_0_–D_3_). The active space contains the same 6 π_in_ and 5 π_out_ orbitals for all the geometries. The DFT-calculated orbitals were used as the initial guess. Harmonic vibration frequencies and zero-point vibrational energies (ZPVEs) were computed at the same level of theory, and all optimized structures were confirmed as true minima. For the geometries optimized at the CASSCF level, energies of the electronic states were computed using CASPT2.^47^ Additional single-point MP2 and AutoCI-CCSD­(T) energies were calculated at the CASSCF-optimized geometries using ROHF orbitals as the reference and def2-TZVP basis set implemented in ORCA.

Magnetically induced ring-current (MIRC) strengths were obtained by integrating the nucleus-independent chemical shielding (NICS) values following the Maxwell–Ampere law.^48^ Separate NICS calculations were carried out at each level of theory, and the resulting NICS values were subsequently used for the corresponding MIRC calculations. At the DFT level, NICS values were computed with Gaussian 16 (revision C.01) using the same exchange–correlation functionals as those employed for geometry optimizations. NICS values at the CASSCF­(11,11) level were calculated with Dalton 2020.1, whereas MP2 NICS values were obtained by using ORCA.

Electron paramagnetic resonance (EPR) parameters of the geometry-optimized structures were calculated at ωB97X-D3 ^49,50^ /EPR-II^51^ level using the ORCA package (version 6.0).^52–54^ The gauge-independent atomic orbital (GIAO) formalism^55,56^ was used to choose the magnetic origin coordinate. The EasySpin package (version 6.0.6)^57^ was used for the simulation of EPR spectra.

Infrared (IR) and Raman spectra of the structure-optimized C_12_N molecules were calculated by ωB97XD/6-311++G** using the Gaussian, and the spectra were visualized by the ChemCraft.

Prior literature of theoretical study on cyclo[18]carbon showed that calculated cumulene- and polyyne-like structures’ consistency with experimental results strongly depends on exchange–correlation functionals.^58^ In this study, we started with use of DFT/ωB97XD/6-311++G­(d,p) method for structural optimization of C_12_N and obtained cumulene- and polyyne-like isomers. After that, we used these optimized geometries as initial structures for subsequent geometric optimizations with other exchange–correlation functionals and methods.

*Molecular Structure, Energy, BLA, BAA, MOs.* After optimization at DFT/ωB97XD/6-311++G­(d,p) level of theory, both molecules show strong distortion at the nitrogen site; thus their structures deviate from the circle-shape. BLA, and bond angle alternation (BAA) are plotted in Figure 1. The cumulene-like structure has highly symmetric bond lengths and angles to the *C*_2*v*_ axis and is 2.19 kcal mol^–1^ more stable than the polyyne-like one (*C*_*s*_ symmetry). Previously, the experimentally obtained 13-membered ring was modeled by DFT as the one with likely with seven double bonds at the opposite side to the nitrogen atom and alternating single and triple for the rest of the C–C bonds^25^ (Supplementary Figure 1), which is considered to be a cumulene-like geometry. The number of π-electrons of the cumulene-like structure is calculated as 13 π_in_ and 14 π_out_ electrons resulting in small paratropic current in π_in_ system and large diatropic current in π_out_ system, leading to diatropic current in total.^25^ In this study, BLA average calculated by ωB97XD for the cumulene-like structure is rather short (0.05 Å). These theoretical results suggest aromaticity of the cumulene-like geometry.

**Figure 1:**
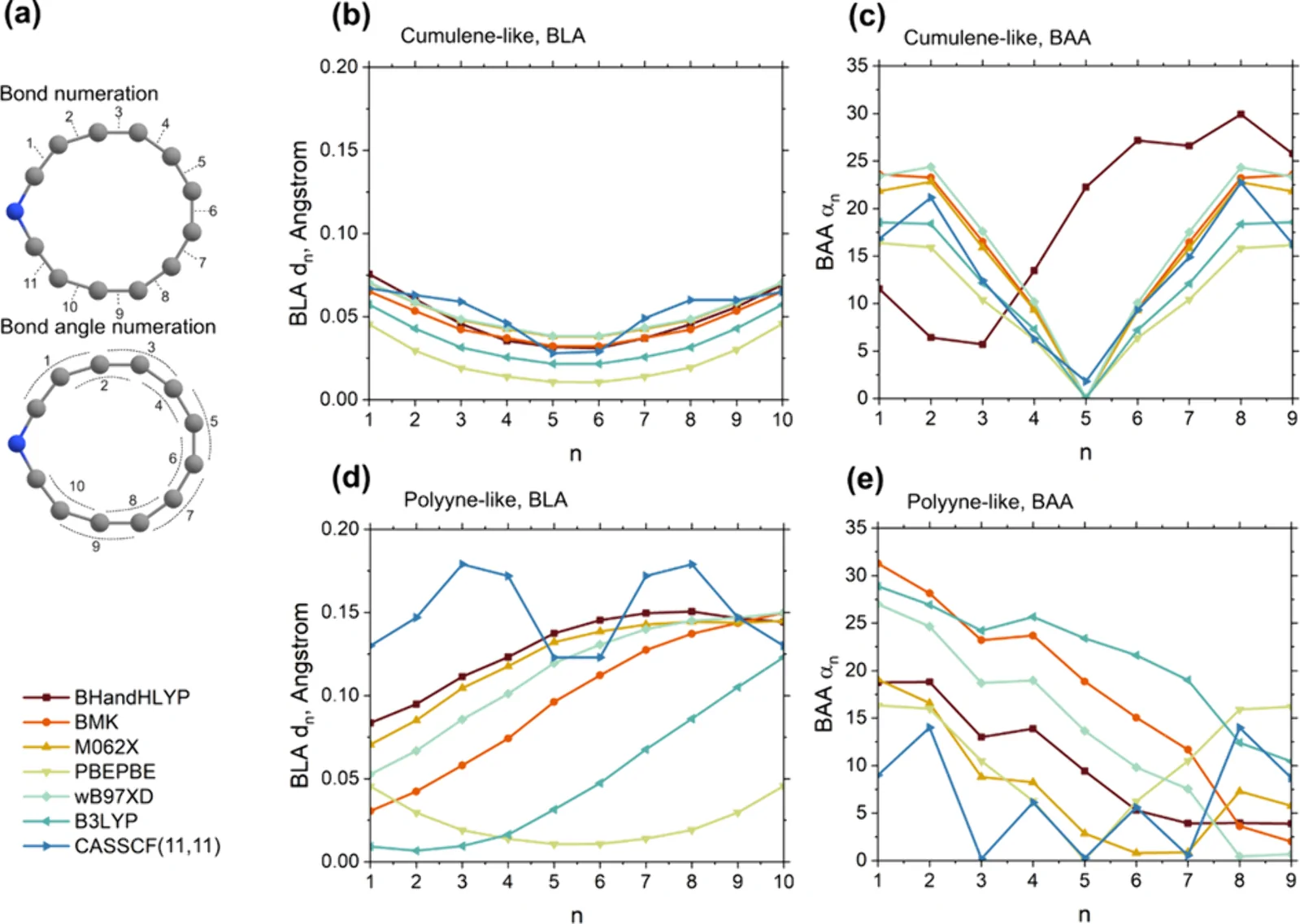
Summary of BLA and BAA. Bond and bond angle numerations are depicted in (a). DFT- and CASSCF­(11,11)-calculated BLA (b, d) and BAA (c, e) of C–C bonds in cumulene- and polyyne-like structures, respectively. Black numbers in the molecular models show bond and angle labels. BLA and BAA are calculated as |*l*_*N*+1_ – *l*_*N*_| where *l* is the bond length or bond angle at the site *N*.

In the case of the polyyne-like geometry, the average BLA calculated by ωB97XD is twice increased (0.11 Å) resulting in a different electronic structure and a decrease of aromaticity.^59,60^ Although the polyyne-like geometry shows asymmetric BAA plot (Figure 1e), its average BAA value (13.5°) is similar to the value of the cumulene-like structure (16.8°). MOs of the polyyne-like geometry are summarized in Supplementary Tables 1 and 2. Analysis of the MOs by their shape shows that the occupied orbitals 1–13 are core orbitals and the next 14–26 orbitals correspond to σ-bonds. Orbitals 27–39 (and 40 for α orbitals) correspond to π bonds where 14 electrons in 28α, 30α, 32α, 34α, 36α, 37α, 40α, 27β, 29β, 31β, 33β, 35β, 37β, 39β are assigned to π_in_ and 13 electrons in 27α, 29α, 31α, 33α, 35α, 38α, 39α, 28β, 30β, 32β, 34β, 36β, 38β are to π_out_. These π electrons cause diatropic current in π_in_ system and paratropic current in π_out_ system, resulting in paratropic current in total.

We further investigate geometries of the C_12_N ring by using several different calculation methods such as DFT with different exchange–correlation functionals (BHandHLYP, BMK, M06-2X, PBEPBE, ωB97XD, B3LYP) or CASSCF. The resulting Cartesian coordinates are summarized in Supplementary Data 1. These calculations confirmed that C_12_N ring has two distinct geometries: cumulene- and polyyne-like structures which slightly differ depending on the type of exchange-correlation functional. The only exception is the results obtained by PBEPBE, for which two structures converged into almost the same cumulene-like geometry. The calculated relative energies (Δ*E*) of cumulene- and polyyne-like structures, defined as Δ*E* = *E*_polyyne_ – *E*_cumulene_, are shown in Table 1. PBE predicts the two structures to be essentially degenerate, whereas all other methods predict the cumulene-like structure to be lower in energy. CASPT2 and CCSD­(T) yield Δ*E* values of 5.03 and 2.71 kcal mol^–1^, respectively, confirming the greater stability of the cumulene-like structure. Calculated absolute values of BLA and BAA are plotted in Figure 1. The BLA values of cumulene-like structures (Figure 1b) are more symmetric than those of polyyne-like structures (Figure 1d) overall. The BAA values of cumulene-like structures are also generally symmetric in relation to the fifth C–C–C angle, contrary to the polyyne-like ones (Figure 1c,e, *n* = 5). We note that DFT and CASSCF also showed different trends in BLA and BAA for cumulene- and polyyne-like C_12_N. For the cumulene-like molecules, DFT predicted smooth and symmetric weak curve of the BLA data points, which suggests smooth drop-shaped ring structures. CASSCF resulted in a more distorted geometry with slightly different BLA tendency from the DFT results even though it is symmetric and the values are within the same range with the DFT results. For BAA, CASSCF showed very similar trend as the DFT results. In the case of the polyyne-like series, DFT showed smooth but asymmetric curve of the BLA data points indicating that the polyyne-type bond alternation is likely localized at bottom half of the molecular model in Figure 1 (around larger value of the angle label). On the other hand, CASSCF predicted a symmetric BAA plot, which is square-wave-like shape at no-N region in the plot. For their BAA, only CASSCF showed zigzag shaped plot where the lower points are almost at 0, indicating that the angles 3 and 4, 5 and 6, and 7 and 8 are almost identical. These theoretical observations indicate that DFT and CASSCF have overall different trends in the optimized geometry of C_12_N, particularly for polyyne-like structures.

**Table 1:** Magnetically Induced Ring-Current Strengths, *I* (nA T^–1^), for the Cumulene- and Polyyne-like C_12_N Structures and the Corresponding Relative Total Electronic Energies, Δ*E* (kcal mol^–1^), Calculated Using DFT, CASSCF, CASPT2, MP2, and CCSD­(T)^a^

	HF exchange (%)	*I*, cumulene-like (nA T^–1^)	*I*, polyyne-like (nA T^–1^)	Δ*E* (kcal mol^–1^)
PBE	0	19.8	19.8	–2.2 × 10^–4^
B3LYP	20	21.3	14.1	6.63
BMK	42	20.9	–19.3	4.68
BHLYP	50	21.4	–9.5	0.52
M062X	54	20.0	–21.8	3.16
ωB97XD	22.2–100	20.8	–11.9	2.19
CASSCF(11,11)		16.8^a^	4.1^a^	–4.09 (5.03)^a^
MP2		20.8^a^	4.9^a^	12.48^a^
CCSD(T)				2.71^a^

a Positive values of Δ*E* indicate that the cumulene-like structure is more stable. For each DFT functional, the two structures were optimized at the corresponding DFT level, and both *I* and Δ*E* were calculated using these optimized geometries. All values obtained with CASSCF, CASPT2, MP2, and CCSD­(T) were calculated using geometries optimized at the CASSCF­(11,11)/def2-TZVP level. The CASPT2 value based on the CASSCF­(11,11) reference wavefunctions is given in parentheses. Ring-current strengths were not calculated at the CCSD­(T) level.

b Calculated using geometries optimized at the CASSCF­(11,11)/def2-TZVP level.

The SA-CASSCF analysis shows that the four lowest electronic states at each geometry are predominantly single-reference doublet states, with each state characterized by a different SOMO. The energetic ordering of these states differs between the two geometries: the D_0_ state of the cumulene-like structure corresponds to D_1_ of the polyyne-like structure, whereas its D_2_ state corresponds to the polyyne-like ground state (D_0_). Despite some differences between the CASSCF and DFT results, both methods identify two C_12_N structures: a more stable and aromatic cumulene-like structure and a less stable and less aromatic polyyne-like structure. Further details, including an energy diagram showing the relative energies and correspondence of these states at the two geometries, are provided in Supplementary Discussion 1.

*Isochemical Shielding Surfaces.* We next analyze aromaticity of two distinct C_12_N isomers by simulating ICSS_*zz*_ surface plots (Figure 2). Positive ICSS_*zz*_ values correspond to magnetic shielding (diatropic response), while negative values indicate deshielding (paratropic response). With ωB97XD, the cumulene-type geometry leads to the diatropic ring current inside the molecule with a weaker paratropic current outside of the ring (Figure 2a). On the other hand, the polyyne-like structure leads to the opposite situation with paratropic current inside the ring and weak diatropic current outside (Figure 2b). This difference in the magnetic response of the two isomers persists for simulations made with different exchange-correlation functionals. Cumulene-like structures simulated by different methods show the consistent shielding inside the ring with the weak deshielding contribution outside of the ring (Figure 2c). On the other hand, simulated polyyne-like structures demonstrated the opposite tendency and the dependence of shielding and deshielding strength on exchange–correlation functionals. Functionals with larger Hartree–Fock exchange contribution result in higher deshielding inside the ring (Figure 2d), while PBE and B3LYP resulted in shielding inside the ring. Finally, the agreement of ICSS_*zz*_ surface plots calculated for the DFT and CASSCF-optimized geometries highlights that the C–N bonds in the polyyne-like structures (the C–N bonds are symmetric in CASSCF and asymmetric in case of DFT simulations) play a small role in the aromaticity of the molecules. These results are also confirmed by AICD simulations summarized in Supplementary Discussion 2.

**Figure 2:**
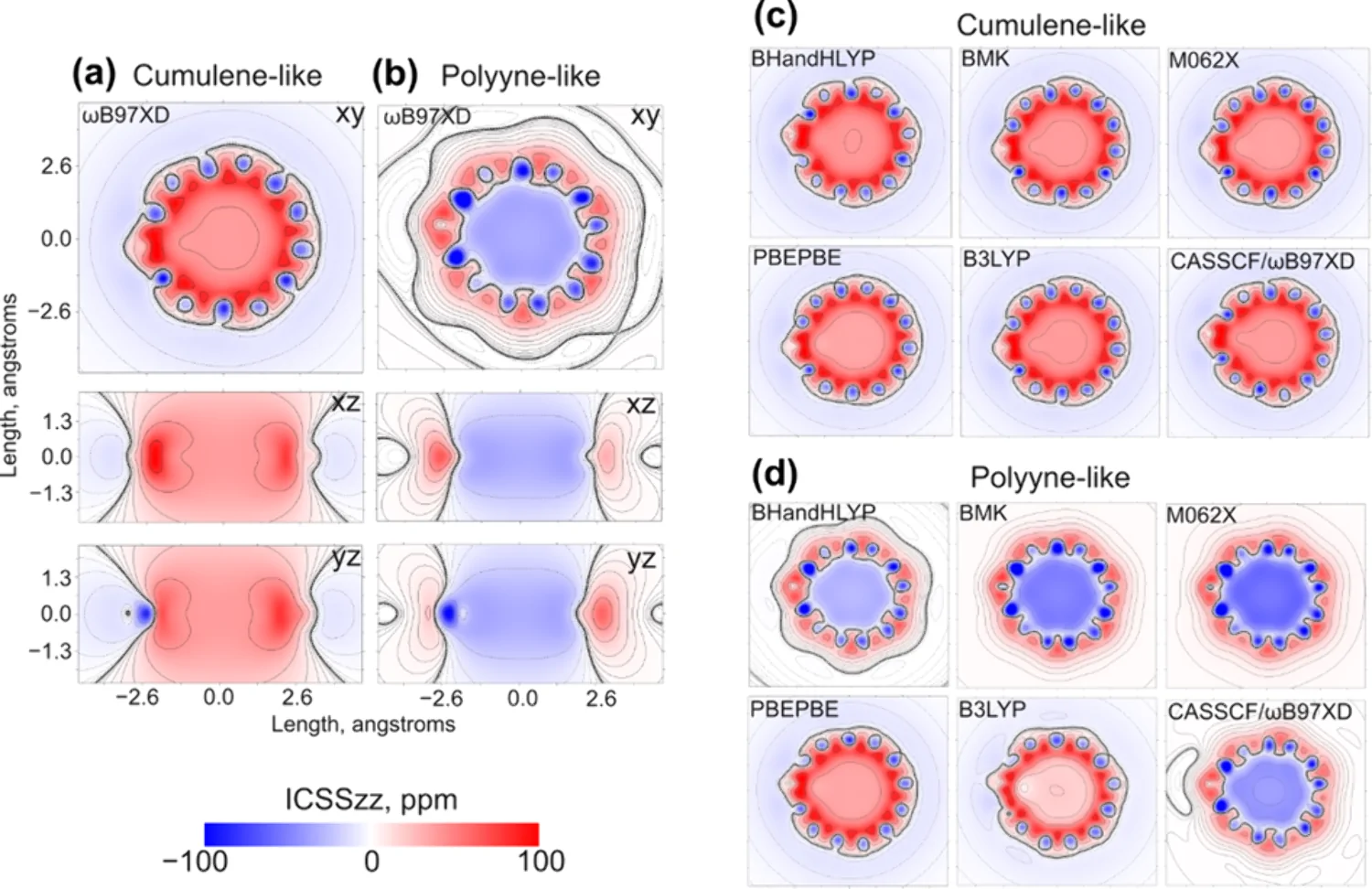
ICSS_*zz*_ mappings of (a) cumulene- and (b) polyyne-like structures calculated by ωB97XD. *XY* projection of ICSS_*zz*_ surface calculated by different methods: BHandHLYP, BMK, M062X, PBEPBE, B3LYP, and CASSCF (ωB97XD single point calculation on CASSCF-optimized geometries) for (c) cumulene-like and (d) polyyne-like geometries. Horizontal and vertical axes show *x*-, *y*-, and *z*-position where C_12_N is positioned at the center. Red and blue colors indicate positive and negative values of chemical shielding (ppm).

*Magnetically Induced Ring Currents.* Magnetically induced ring current (MIRC) strengths calculated by different methods are summarized in Table 1. The cumulene-like structure is strongly aromatic regardless of the level of theory. The MIRC strength of the cumulene-like structure varies from 16.8 nA T^–1^ at the CASSCF level to 21.4 nA T^–1^ with BHLYP. These values are close to the previously reported value of 21 nA T^–1^ for C_12_N.^25^ Aromaticity can be discussed based on the net ring current with the value for a benzene molecule (11.8 nA T^–1^) as a reference.^61,62^

In turn, for the polyyne-like structure, the MIRC strength strongly depends on the level of theory. By increasing Hartree–Fock exchange in the exchange-correlation functional, the MIRC strength nonmonotonically changes from a strongly diatropic value of 19.8 nA T^–1^ for PBE to a strongly paratropic value of −21.8 nA T^–1^ for M062X. With MP2, which accounts for dynamic electron correlation, and CASSCF, which accounts for static correlation, the MIRC remains weakly diatropic, with strengths of 4.9 nA T^–1^ and 4.1 nA T^–1^, respectively.

These results show the aromaricity of the cumulene-like C_12_N and the antiaromaticity or low aromaticity of the polyyne-like C_12_N. These observations are comparable to the case of cyclo[18]­carbon, in which the cumulene-like structure exhibits twice the larger net diatropic ring current than the polyyne-like one.^58^

*Unpaired Electron Distribution and EPR Analysis.* We next investigate unpaired electron by DFT/ωB97XD method. Although the unpaired electron is distributed throughout the ring in both cases, the two distinct isomers have different spin-density distribution (Figure 3). For the cumulene-like structure, the unpaired electron spin is symmetrically spread on π_in_-plane on the ring as shown in Figure 3a. In the case of the polyyne-like molecule, the spin is delocalized asymmetrically, mostly spread to π_out_ direction (Figure 3b). The positive- and negative-signed spin density regions, which are plotted by (α-spin distribution) – (β-spin distribution), are more visible in the polyyne-like structure due to the different skew of α and β spin distributions.

**Figure 3:**
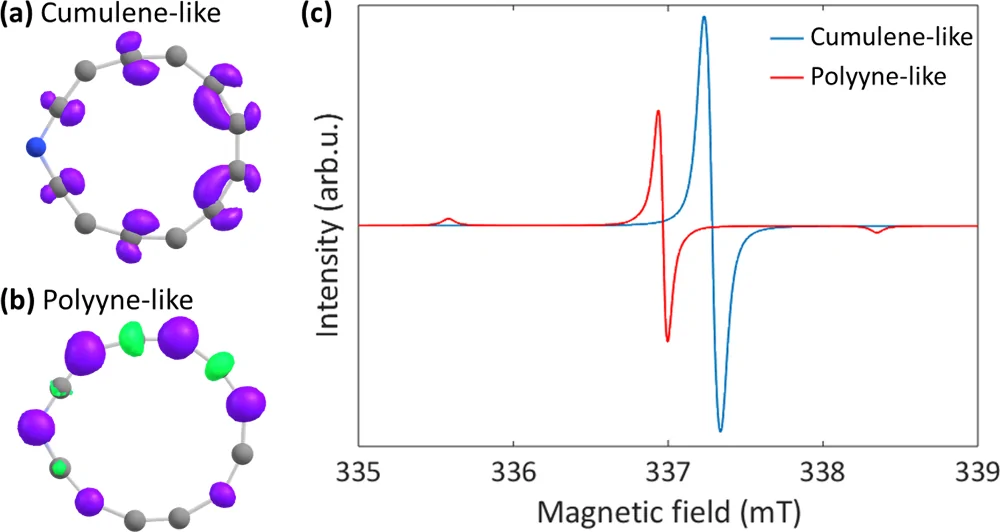
DFT-optimized (a) cumulene- and (b) polyyne-like structures and (c) their EPR spectra. For (a) and (b), gray and blue balls represent carbon and nitrogen atoms, respectively. Purple and green bubble plots show signed spin density with an isosurface cutoff value set to 0.01 e a_0_^–3^. (c) is simulated by using the DFT-calculated isotropic *g*-values and anisotropic nitrogen hyperfine coupling constants *A* ^N^. Microwave frequency is set to 9.45 GHz, and full width at half-maximum (fwhm) of Lorentzian-broadening is set to 0.1 mT.

Subsequently, we calculate electronic *g*-factor, which also can be used for discussion of aromaticity. By the DFT calculations, we obtain *g*-tensors; *g*_*x*_ = 2.0006, *g*_*y*_ = 2.0023, *g*_*z*_ = 2.0025, and *g*_iso_ = 2.0018 for the cumulene-like structure, and *g*_*x*_ = 2.0023, *g*_*y*_ = 2.0037, *g*_*z*_ = 2.0050, and *g*_iso_ = 2.0037 for the polyyne-like structure. The polyyne-like structure shows anisotropic *g*-values (*g*_*x*_ ≠ *g*_*y*_ ≠ *g*_*z*_), which comes from the planar cyclic system and asymmetric electron distribution due to the alternating bond lengths. In contrast, the cumulene-like molecule shows axial *g*-anisotropy (*g*_*y*_ = *g*_*z*_). This can be attributed to the almost symmetrically delocalized unpaired electron, where only small amount of spin is located on the nitrogen. Δ*g* between the two conformations might be distinguishable, e.g., by STM-EPR. The ring current of the cyclic system affects the effective magnetic field, and thus molecules with stronger aromaticity have smaller *g*-values due to the resonance at lower magnetic field.^63^ Therefore, the cumulene-like molecule is more aromatic than the polyyne-like one, consistent with the observations from BLA, BAA, and ICSS_*zz*_ investigations.

We also investigate hyperfine coupling parameters (*A*). DFT-calculated *A* values for the nitrogen atoms are *A* ^N^_*x*_ = −0.8, *A* ^N^_*y*_ = 0.2, *A* ^N^_*z*_ = −1.7, and *A* ^N^_iso_ = 0.4 for the cumulene-like molecule and are *A* ^N^_*x*_ = 38.8, *A* ^N^_*y*_ = 1.0, *A* ^N^_*z*_ = 0.5, and *A* ^N^_iso_ = 13.4 for the polyyne-like molecule. The cumulene-like structure possesses very small amount of spin distribution on the nitrogen atom, and this results in the low *A* ^N^ values. In the case of the polyyne-like molecule, the spin density is located at the nitrogen site with a π_out_ shape. These result in the axial and larger *A* value, which would also be an experimental fingerprint to distinguish the polyyne-like structure from the cumulene-like one. Simulated EPR spectra of the cumulene- and polyyne-like structures generated using the DFT-calculated isotropic *g*-values and anisotropic *A* ^N^ values are shown in Figure 3. The spectrum of the cumulene-like structure shows a single peak, which contains only negligible amounts of the nitrogen component due to the slight density of spin on the nitrogen site (Figure 3a), while the polyyne-like geometry exhibits additional two small peaks, which are remnants of the nitrogen characteristic triple peak distorted by the *A* anisotropy. Nevertheless, these overall peak shapes would be very similar under experimental conditions. Therefore, possible experimental identification of the cumulene- and polyyne-like structures from their EPR spectra would depend on relative *g*-value.

*Vibrational Spectra.* DFT/ωB97XD-calculated IR and Raman spectra of the cumulene- and polyyne-like isomers are shown in Figure 4. For the IR signals (Figure 4a,b), the cumulene-like structure with higher symmetry exhibits clear prominent peaks at 1713 cm^–1^, 1856 cm^–1^, 2147 cm^–1^, and 2214 cm^–1^, which correspond to C=C stretching mode.^64,65^ In the polyyne-like one, the intensity magnitude of most peaks is low, which is a consequence of its low molecular symmetry. The peaks gathered at higher frequency (2200–2000 cm^–1^) correspond to C≡C stretching modes.^64,66,67^ The peaks at 1237 cm^–1^ and 1516 cm^–1^ from the cumulene-like structure and the ones at 1139 cm^–1^ and 1607 cm^–1^ from the polyyne-like one originate from CN stretching modes.

**Figure 4:**
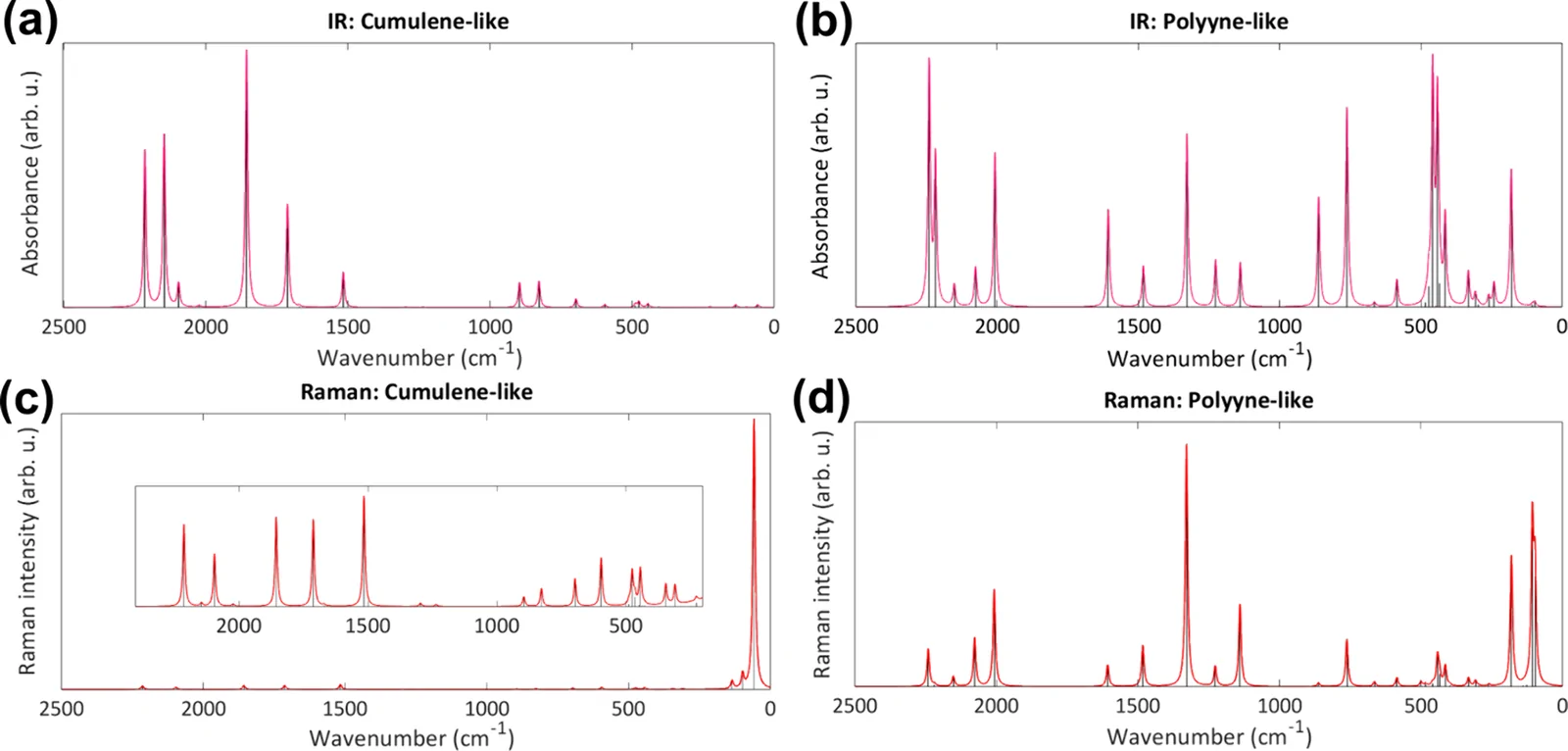
DFT/ωB97XD-calculated IR (a, b) and Raman (c, d) spectra of the cumulene- and polyyne-like structures. Raman spectra were calculated with the incidence wavelength of 514 nm and temperature of 298.15 K. Black and pink/red lines are before and after Lorentzian line-broadening with full width at half-maximum (fwhm) set to 10 cm^–1^, respectively. Inset in (c) is ×200 enlarged along intensity.

Raman scattering activity spectra and Raman intensity spectra simulated for an incidence wavelength of 514 nm at room temperature are shown in Supplementary Figure 2 and Figure 4c,d, respectively. In the Raman scattering activity spectra (Supplementary Figure 1), the cumulene-like structure shows 5 prominent peaks which correspond to CC stretch modes (seen throughout 1200–2200 cm^–1^), whereas the polyyne-like structure exhibits a larger number of discernible CC stretch peaks with various intensities in 1200–2200 cm^–1^ due to the low molecular symmetry. The spectral features in the higher-frequency region are particularly similar to the reported ones of cumulenic and polyynic linear molecules, respectively.^68^ The resulting Raman intensity spectra (Figure 4c,d) exhibit notably different overall spectroscopic features between the cumulene- and polyyne-like structures. A strong peak at very low frequency is observed in the spectrum of the cumulene-like structure, while the polyyne-like one keeps higher-frequency peaks visible.

We carried out a theoretical investigation on two structural isomers of the C_12_N ring to complement the previously reported cumulene-like isomer^25^ and add insights into another isomer, shaped as a polyyne-like structure.

Aromaticity of the cumulene- and polyyne-like structures was discussed through the molecular structure (BLA and BAA), π-electron distribution (ring current), and *g*-factors. The two conformations exhibited opposite features through all these parameters, consistently suggesting that the cumulene-like molecule is aromatic while the polyyne-like one is antiaromatic/less-aromatic.

Calculations with a higher level of theory showed a different degree of aromaticity depending on methods, indicating the high sensitivity of C_12_N structures to the choice of exchange-correlation.

Despite their different parameters, the two conformations are similar in energy (difference of 2.19 kcal mol^–1^ with ωB97XD). Therefore, both of them would be experimentally reachable by controlling conditions. We provide calculated EPR parameters for identifying them by examining their EPR features. In addition, our simulations suggest that the two isomers can also be distinguished experimentally by IR or Raman spectra.

## Supplementary Material



## Notes

Supplementary Figure 1 showing molecular structures based on ωB97XD; Supplementary Figure 2 showing DFT-calculated Raman scattering activity; Supplementary Table 1 listing molecular orbitals of the polyyne-like structure (α-spin); Supplementary Table 2 listing molecular orbitals of the polyyne-like structure (β-spin); Supplementary Discussion 1 on electronic states of the cumulene- and polyyne-like C_12_N; Supplementary Discussion 2 on AICD simulations; and Supplementary Data 1 listing Cartesian coordinates of optimized C_12_N structures (PDF)
