# Supplementary material for: Electronic Structure and Spectral Properties of Radical Heterocyclocarbon C12N Isomers
Source: J Phys Chem Lett. 2026 Aug 25;17(36):10332–9. doi: 10.1021/acs.jpclett.6c01817 (PMC13573620; doi:10.1021/acs.jpclett.6c01817)
Supplement: Supplementary file 1 [file jz6c01817_si_001.pdf]

Electronic structure and spectral properties of radical hetero-cyclocarbon $C_{12}N$ isomers

Yuri Tanuma,[a] Ihor Sahalianov,[a,b] Rinat Nasibullin, and Glib Baryshnikov[a,b,*]

a) Laboratory of Organic Electronics, Department of Science and Technology, Linköping University, SE-60174 Norrköping, Sweden.

b) Wallenberg Initiative Materials Science for Sustainability, Department of Science and Technology, Linköping University, SE-60174 Norrköping, Sweden.

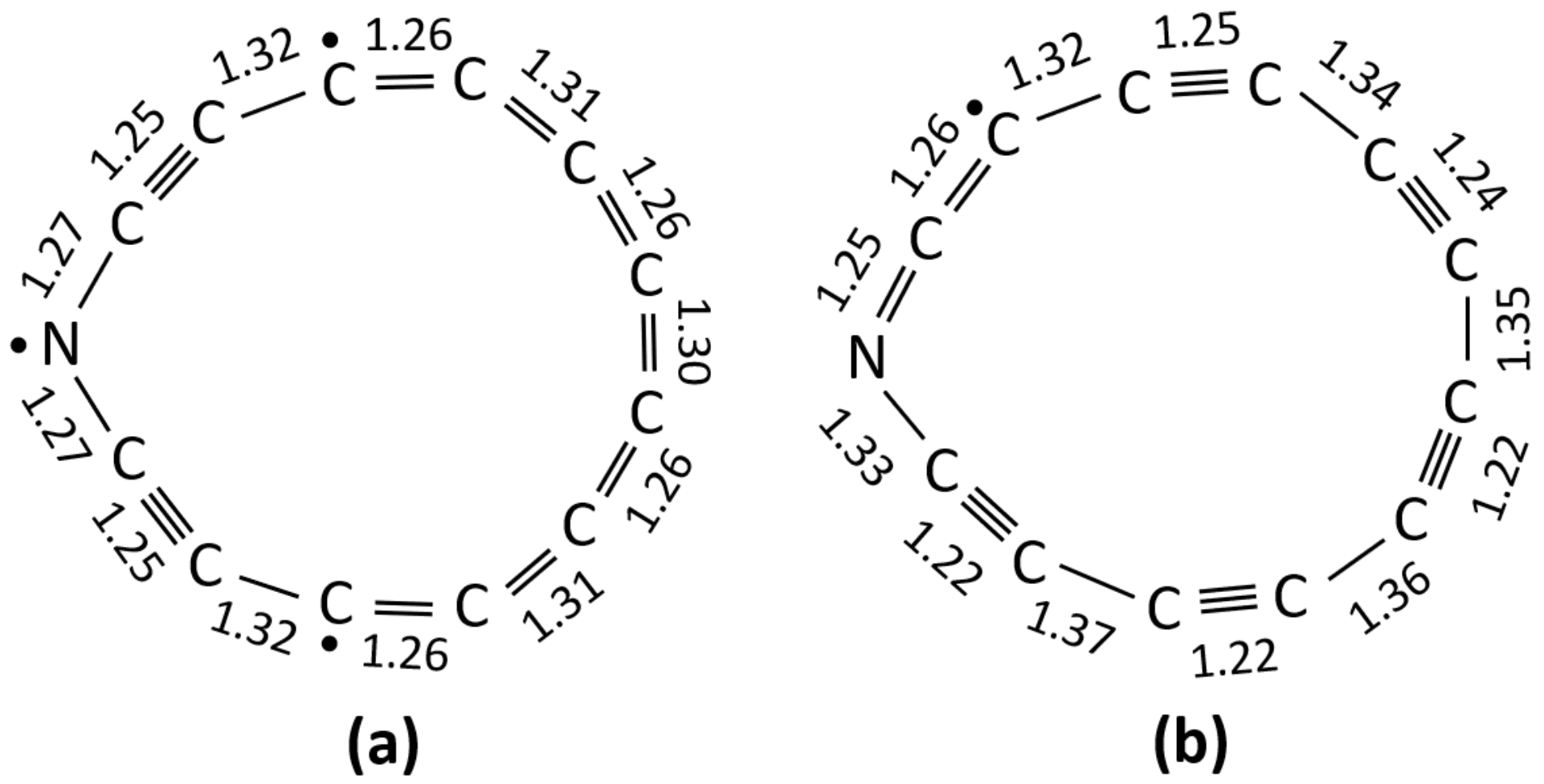

Supplementary Figure 1. Molecular structures of (a) cumulene-like and (b) polyyne-like $C_{12}N$ depicted based on DFT (ωB97XD) results. Values on bonds indicate bong lengths in Å.

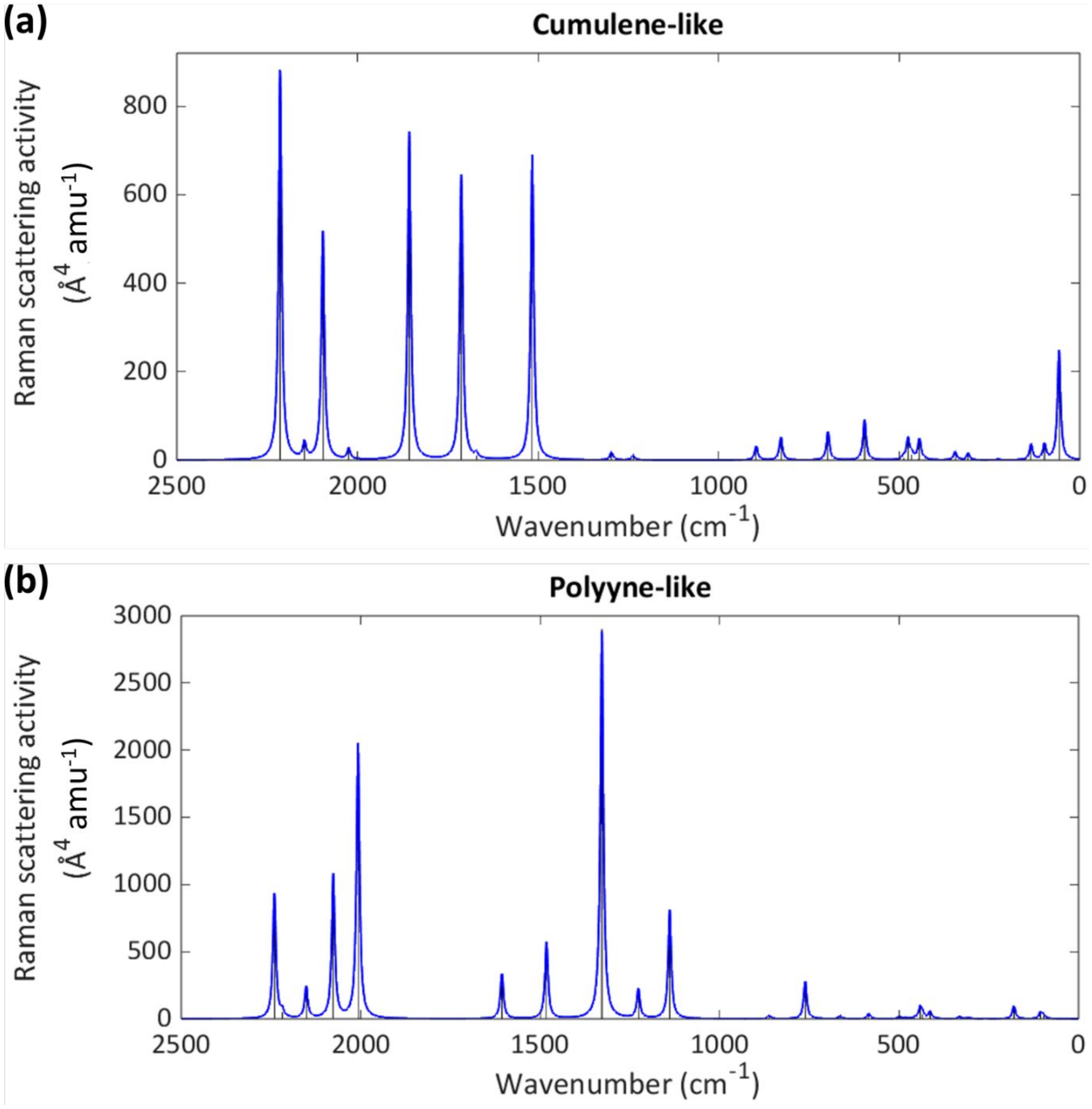

Supplementary Figure 2. DFT-calculated Raman scattering activity of the (a) cumulene- and (b) polyyne-like structures. Black and blue lines are before and after Lorentzian line-broadening with full width at half maximum (FWHM) set to 10 cm^{-1}, respectively.

Supplementary Table 1. Molecular orbitals of the polyyne-like structure (α-spin). Grey and blue spheres represent carbon and nitrogen atoms, respectively. Bubble plot represents signed molecular orbitals. Isosurface cut-off is set at 0.03 e a_0^{-3}.

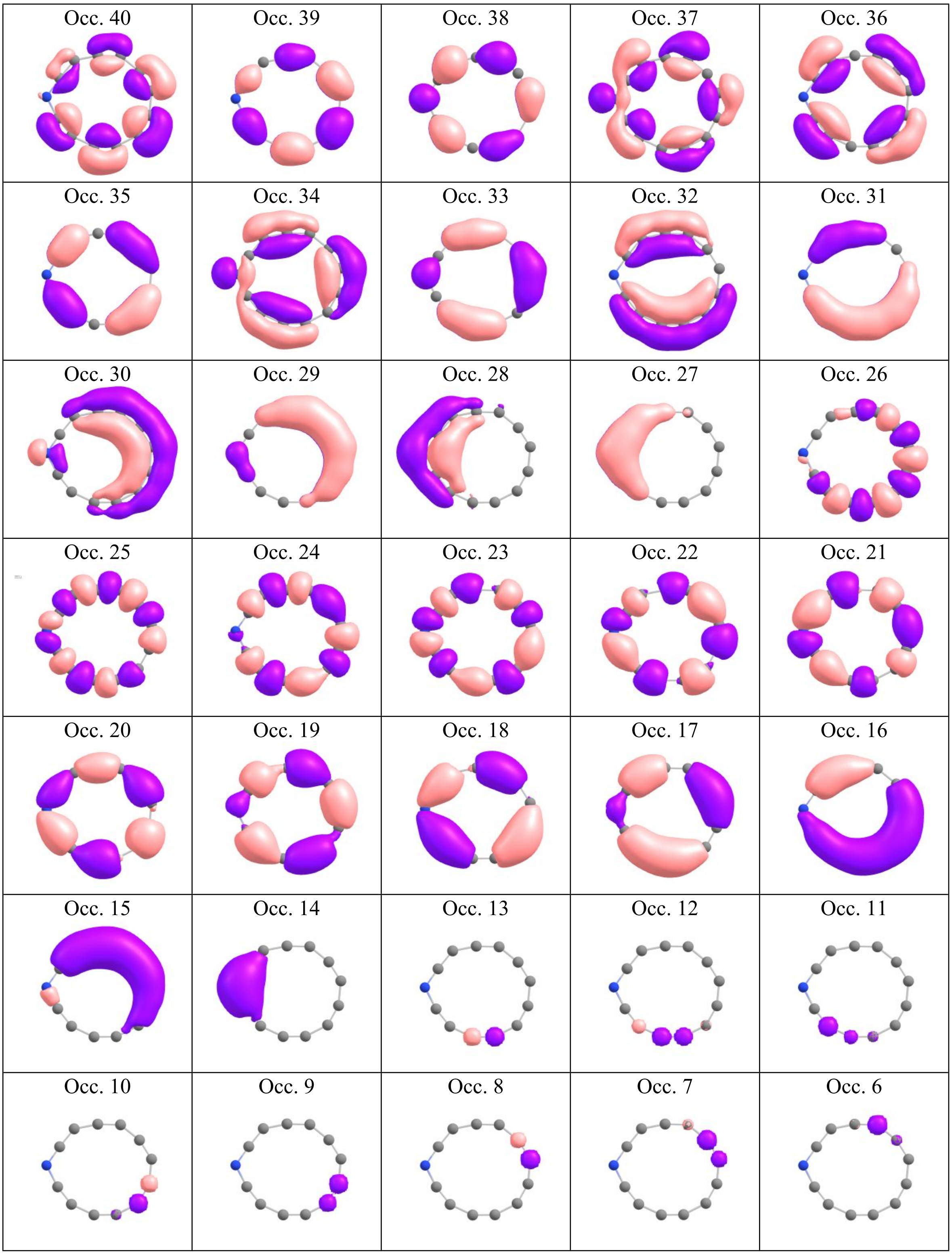

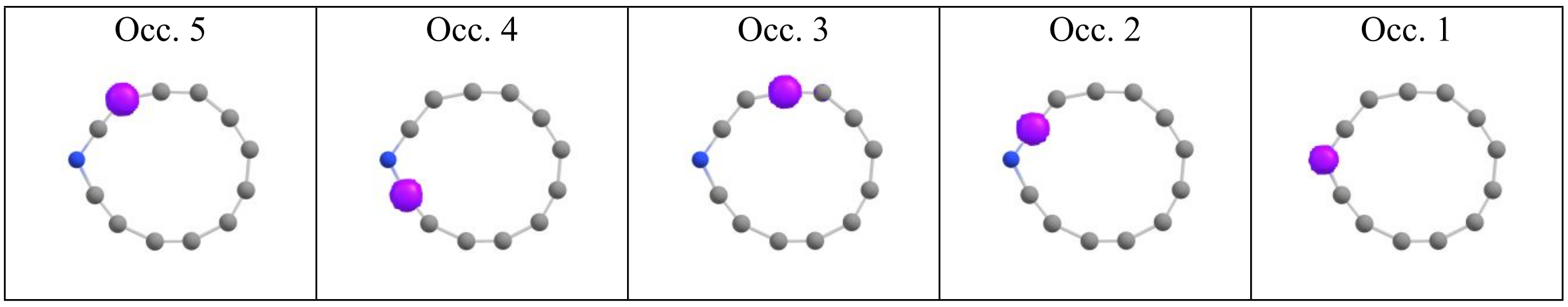
Occ. 5
Occ. 4
Occ. 3
Occ. 2
Occ. 1

Supplementary Table 2. Molecular orbitals of the polyyne-like structure (β-spin). Grey and blue spheres represent carbon and nitrogen atoms, respectively. Bubble plot represents signed molecular orbitals. Isosurface cut-off is set at 0.03 e a_0^{-3}.

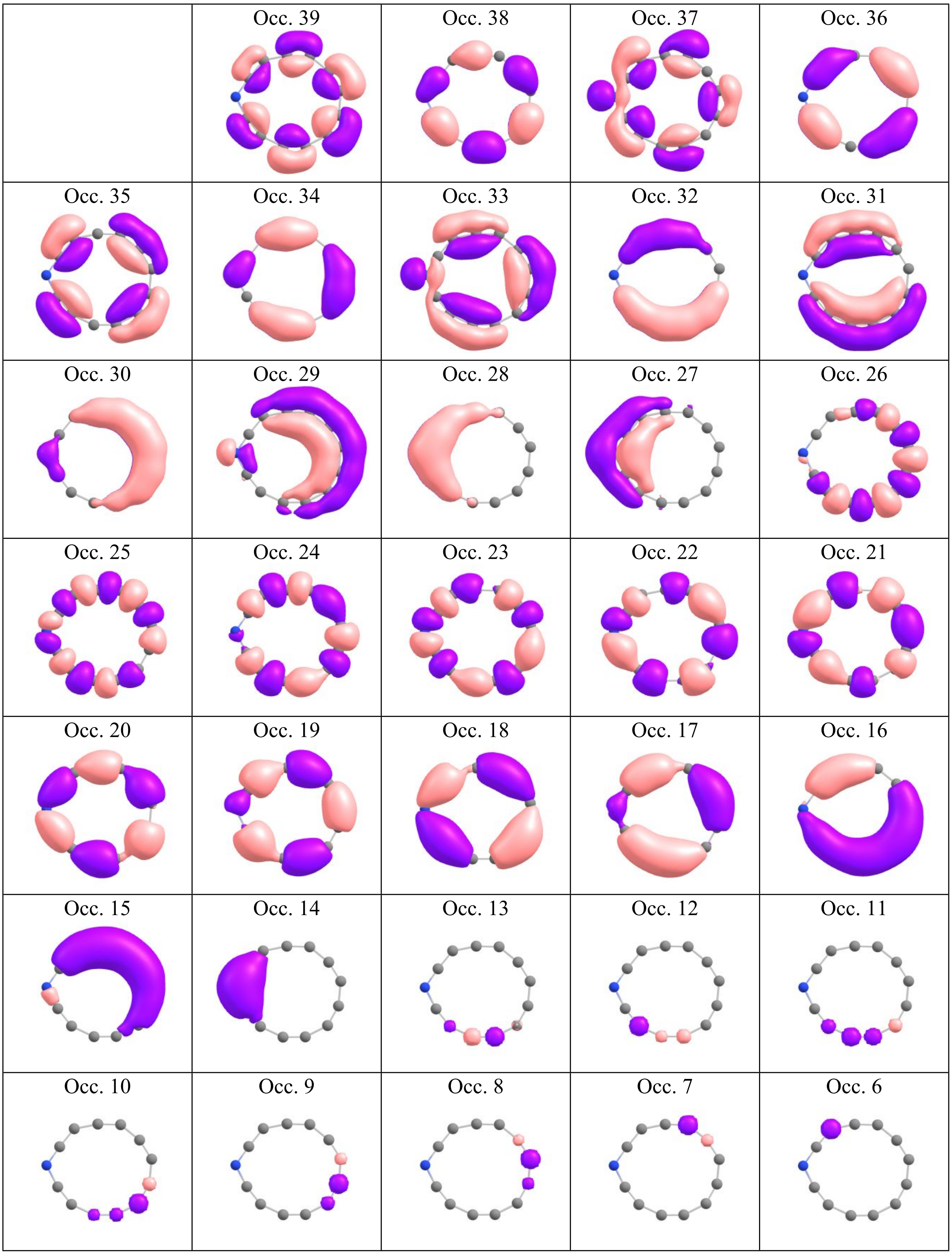

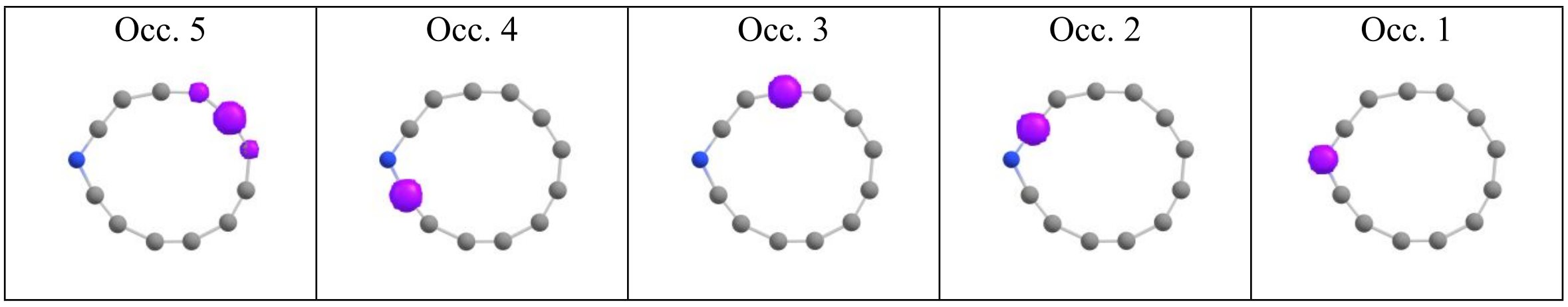
Occ. 5
Occ. 4
Occ. 3
Occ. 2
Occ. 1

Supplementary Discussion 1. Electronic states of the cumulene- and polyene-like $C_{12}N$

The aromatic, cumulene-like structure is more stable than the less aromatic, polyyne-like structure by 2.12 kcal mol^{-1}, based on the CASPT2 electronic-energy difference including ZPVE corrections calculated at the CASSCF(11,11) level (**Supplementary Table D1**). Based on this energy difference, the estimated Boltzmann population of the polyyne-like structure at 298 K is only 2.7%. Each structure is associated by its own doublet ground state. As illustrated in **Supplementary Figure D1**, each doublet electronic state is characterised by a distinct SOMO: SOMO of the cumulene-like structure is π-in, while SOMO of the polyyne-like structure is π-out. The order of the D_0, D_1, and D_2 states of the cumulene- and polyyne-like structures are different, while D_3 remains. The electronic state D_0 at the cumulene-like geometry becomes D_1 at the cumulene-like geometry, whereas the D_2 state at the cumulene-like geometry becomes the ground doublet state at the polyyne-like geometry.

Supplementary Table D1. Relative energies including ZPVE (ΔE in kcal mol^{-1}, referenced to the energy of D_0 of the polyyne-like structure), population at 298 K (P), vertical excitation energies ($E(D_n$-$D_0)$ in kcal mol^{-1}, n = 1-3), and magnetically induced ring-current strengths (I in nA T^{-1}) for (a) cumulene- and (b) polyyne-like structures.

	(a) Cumulene-like	(b) Polyyne-like
ΔE	0.0	+2.12
P	97.3%	2.7%
$E(D_1$-$D_0)$	5.24	13.84
$E(D_2$-$D_0)$	7.78	19.55
$E(D_3$-$D_0)$	23.94	21.87
I	16.77	4.06

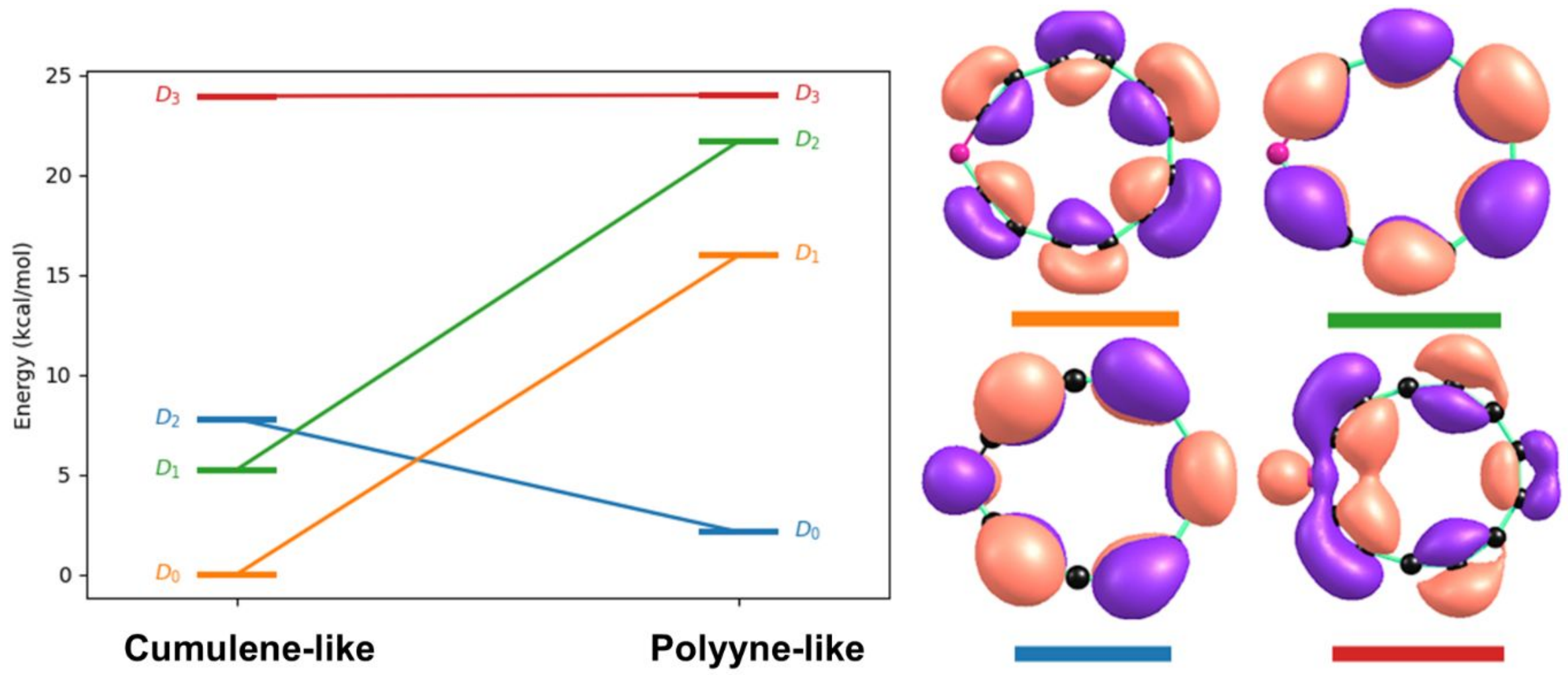

Supplementary Figure D1. Diagram of the four lowest doublet states D_n (n=0-3) for the cumulene- and polyyne-like structures (left). Horizontal ticks mark the excitation energies of each state, referenced to cumulene-like D_0 energy. Connecting lines link corresponding states between the two geometries based on CASSCF(11,11) CI root tracking. The singly occupied MOs (right) associated with each root are shown in the corresponding color.

Supplementary Discussion 2. AICD simulations

We calculated anisotropy of the induced current density (AICD)[40] of two $C_{12}N$ conformations with different methods. Obtained total AICD plots are shown in **Supplementary Figure D2**. The cumulene-like geometries (**Supplementary Figure D2a**) exhibit diatropic current while the polyyne-like ones (**Supplementary Figure D2b**) have paratropic current (with the exception of PBE and B3LYP results), indicating their aromaticity and anti-aromaticity, respectively. These results confirm $ICSS_{zz}$ simulations and supplement it with detailed current vectors mapping.

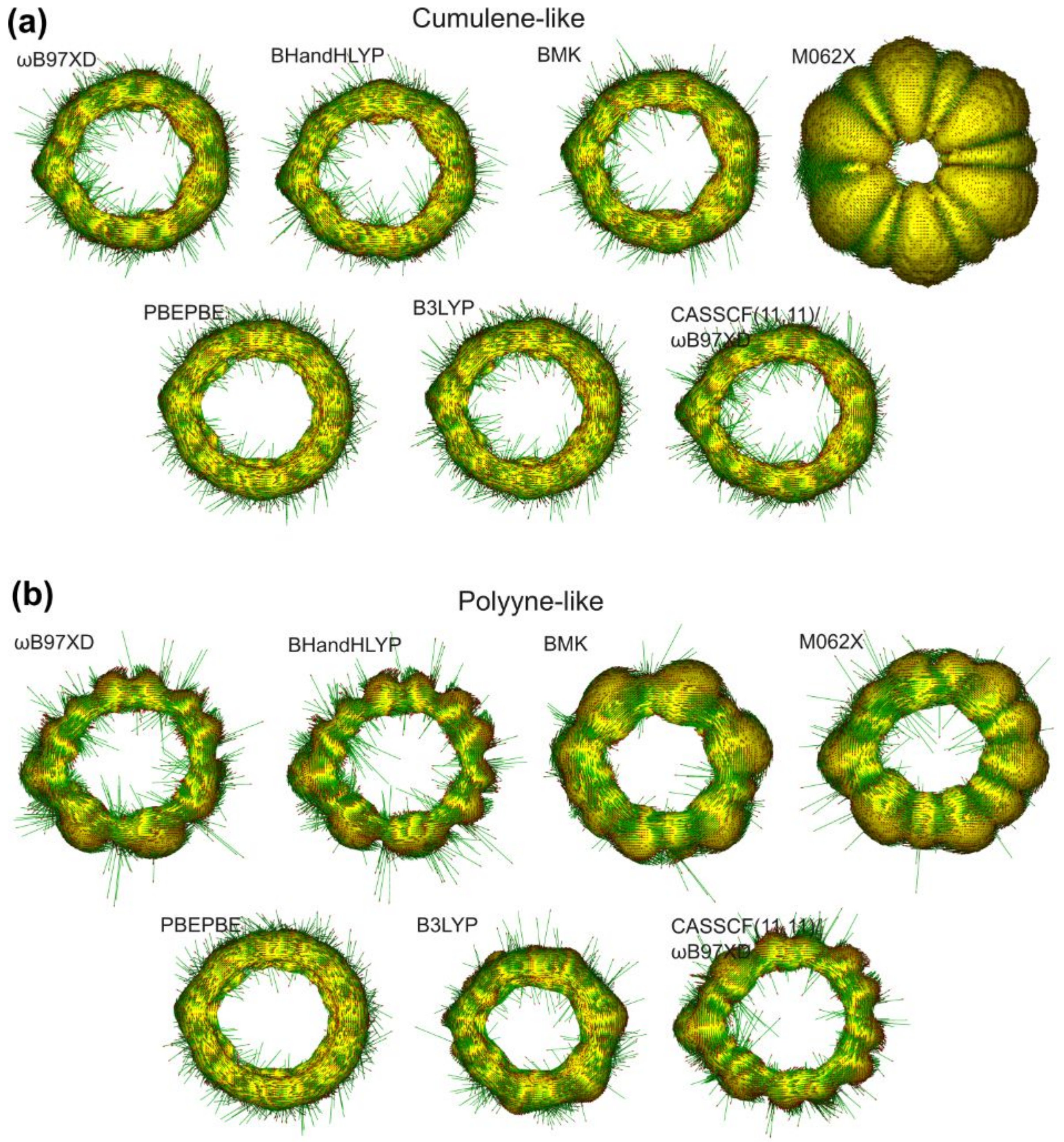

Supplementary Figure D2. Total AICD plots of (a) cumulene- and (b) polyyne-like geometries calculated by different methods: BHandHLYP, BMK, M062X, PBEPBE, B3LYP, and CASSCF (ωB97XD single point calculation on CASSCF-optimised geometries). Green lines with red arrowhead show anisotropic current vector, and yellow bubble plots show isosurface of the current density. The magnetic field vector is perpendicular to the paper/screen, pointing toward readers.

Supplementary Data 1. Cartesian coordinates (Å) of $C_{12}N$ optimised with different methods.

ωB97XD/6-311++G(d,p)

Cumulene-like

N	-2.876280	-0.302521	0.000000
C	-2.355848	0.859498	0.000000
C	-1.736361	1.939497	0.000000
C	-0.518685	2.437496	0.000000
C	0.726843	2.605729	0.000000
C	1.843494	1.929872	0.000000
C	2.610891	0.928185	0.000000
C	2.747393	-0.364848	0.000000
C	2.204595	-1.503989	0.000000
C	1.253315	-2.397745	0.000000
C	0.000000	-2.491873	0.000000
C	-1.294652	-2.258162	0.000000
C	-2.125325	-1.330719	0.000000

Polyyne-like

C	1.717650	-1.827253	0.000000
C	0.520863	-2.484374	0.000000
C	0.000000	2.455684	0.000000
C	-0.695986	-2.547492	0.000000
C	-1.948470	-2.008557	0.000000
C	-2.822874	0.356711	0.000000
C	-2.613235	-0.981245	0.000000
C	-2.228952	1.439265	0.000000
C	-1.245043	2.342839	0.000000
C	1.305477	2.282539	0.000000
C	2.410388	-0.828510	0.000000
C	2.162916	1.353544	0.000000
N	2.946228	0.383014	0.000000

PBE/6-311++G(d,p)

Cumulene-like

N	-2.877320	-0.315217	0.000000
C	-2.154911	-1.367241	0.000000
C	-1.284366	-2.285409	0.000000
C	0.000000	-2.546632	0.000000
C	1.271120	-2.387283	0.000000
C	2.242051	-1.522571	0.000000
C	2.768352	-0.348964	0.000000
C	2.628287	0.940295	0.000000
C	1.859709	1.971775	0.000000
C	0.724677	2.606095	0.000000
C	-0.550589	2.486922	0.000000
C	-1.747536	1.952347	0.000000
C	-2.399921	0.868418	0.000000

Polyyne-like

C	1.747262	-1.953217	0.000000
C	0.550387	-2.487538	0.000000
C	0.000000	2.546948	0.000000
C	-0.725045	-2.607144	0.000000
C	-1.859366	-1.971695	0.000000
C	-2.768195	0.349970	0.000000
C	-2.626683	-0.939370	0.000000
C	-2.241875	1.523725	0.000000
C	-1.271309	2.388842	0.000000
C	1.284177	2.284813	0.000000
C	2.399602	-0.869227	0.000000
C	2.155105	1.366994	0.000000
N	2.876520	0.314485	0.000000

M062X/6-311++G(d,p)

Cumulene-like

N	-2.893802	-0.305358	0.000000
C	-2.367828	0.855375	0.000000
C	-1.736090	1.929982	0.000000
C	-0.520122	2.435643	0.000000
C	0.727824	2.598204	0.000000
C	1.853417	1.935023	0.000000
C	2.621185	0.931051	0.000000
C	2.758259	-0.363631	0.000000
C	2.216761	-1.505663	0.000000
C	1.254451	-2.389312	0.000000
C	0.000000	-2.490210	0.000000
C	-1.294703	-2.249542	0.000000
C	-2.137051	-1.330668	0.000000

Polyyne-like

C	1.725483	-1.833335	0.000000
C	0.510783	-2.455322	0.000000
C	0.000000	2.468323	0.000000
C	-0.706040	-2.554458	0.000000
C	-1.942515	-1.975489	0.000000
C	-2.813191	0.365785	0.000000
C	-2.661848	-0.986932	0.000000
C	-2.271805	1.469113	0.000000
C	-1.234085	2.327340	0.000000
C	1.307168	2.238230	0.000000
C	2.419274	-0.830051	0.000000
C	2.187209	1.341063	0.000000
N	2.982485	0.364913	0.000000

BMK/6-311++G(d,p)

Cumulene-like

N	-2.852186	-0.304744	0.000000
C	-2.359381	0.864785	0.000000
C	-1.748287	1.955898	0.000000
C	-0.530149	2.453269	0.000000
C	0.722150	2.612118	0.000000
C	1.840769	1.940759	0.000000
C	2.608520	0.932219	0.000000
C	2.746866	-0.360144	0.000000
C	2.208941	-1.507852	0.000000
C	1.257623	-2.400626	0.000000
C	0.000000	-2.509710	0.000000
C	-1.295787	-2.281241	0.000000
C	-2.123715	-1.343942	0.000000

Polyyne-like

C	1.724839	-1.843753	0.000000
C	0.536819	-2.519633	0.000000
C	0.000000	2.469522	0.000000
C	-0.685881	-2.555010	0.000000
C	-1.940934	-2.030108	0.000000
C	-2.821845	0.347151	0.000000
C	-2.581097	-0.976322	0.000000
C	-2.206480	1.434005	0.000000
C	-1.260268	2.358937	0.000000
C	1.299811	2.328650	0.000000
C	2.412307	-0.839422	0.000000
C	2.143140	1.369891	0.000000
N	2.896791	0.390937	0.000000

BHandHLYP/6-311++G(d,p)

Cumulene-like

N	2.907865	0.305291	0.000000
C	2.385499	-0.856361	0.000000
C	1.717291	-1.895117	0.000000
C	0.523071	-2.435301	0.000000
C	-0.725330	-2.500131	0.000000
C	-1.901401	-1.956183	0.000000
C	-2.563273	-0.883679	0.000000
C	-2.824102	0.381754	0.000000
C	-2.181396	1.466602	0.000000
C	-1.251328	2.371934	0.000000
C	0.000000	2.426367	0.000000
C	1.291014	2.214439	0.000000
C	2.137446	1.309505	0.000000

Polyyne-like

C	1.711091	-1.828834	0.000000
C	0.503601	-2.449153	0.000000
C	0.000000	2.451016	0.000000
C	-0.705200	-2.522713	0.000000
C	-1.947289	-1.964906	0.000000
C	-2.816712	0.381503	0.000000
C	-2.629062	-0.962847	0.000000
C	-2.248429	1.460941	0.000000
C	-1.225755	2.331495	0.000000
C	1.304365	2.210140	0.000000
C	2.410693	-0.837582	0.000000
C	2.179307	1.327388	0.000000
N	2.968620	0.345902	0.000000

B3LYP/6-311++G(d,p)

Cumulene-like

N	-2.873866	-0.309709	0.000000
C	-2.380698	0.863468	0.000000
C	-1.738624	1.938854	0.000000
C	-0.535974	2.457632	0.000000
C	0.723821	2.591424	0.000000
C	1.853066	1.950630	0.000000
C	2.617336	0.932954	0.000000
C	2.756296	-0.353938	0.000000
C	2.226012	-1.510918	0.000000
C	1.259412	-2.377802	0.000000
C	0.000000	-2.515310	0.000000
C	-1.285550	-2.264657	0.000000
C	-2.142254	-1.351008	0.000000

Polyyne-like

C	1.703626	-1.851058	0.000000
C	0.555235	-2.556296	0.000000
C	0.000000	2.494059	0.000000
C	-0.687171	-2.532403	0.000000
C	-1.924722	-2.048768	0.000000
C	-2.810563	0.330883	0.000000
C	-2.543856	-0.950119	0.000000
C	-2.186164	1.444793	0.000000
C	-1.277333	2.364977	0.000000
C	1.281220	2.339389	0.000000
C	2.427386	-0.863266	0.000000
C	2.131666	1.381038	0.000000
N	2.854865	0.382946	0.000000

CASSCF(11,11)/def2-TZVP

Cumulene-like

N	2.967307	0.315578	-0.000049
C	2.387879	-0.821910	-0.000008
C	1.684547	-1.837710	0.000016
C	0.494779	-2.363657	0.000016
C	-0.732377	-2.550989	0.000042
C	-1.867645	-1.915888	-0.000030
C	-2.634430	-0.925744	0.000012
C	-2.755599	0.349976	-0.000060
C	-2.224034	1.485084	0.000016
C	-1.238577	2.331755	-0.000015
C	-0.001744	2.418525	0.000057
C	1.275545	2.159188	0.000027
C	2.167901	1.304677	-0.000023

Polyyne-like

N	3.011638	0.346341	0.000000
C	2.428195	-0.799858	0.000000
C	1.715391	-1.789695	0.000000
C	0.508539	-2.394780	0.000000
C	-0.682370	-2.562247	0.000000
C	-1.938647	-1.986218	0.000000
C	-2.596521	-0.970539	0.000000
C	-2.750329	0.353873	0.000000
C	-2.337459	1.491405	0.000000
C	-1.246825	2.339569	0.000000
C	-0.048873	2.447477	0.000000
C	1.262914	2.130643	0.000000
C	2.183309	1.330194	0.000000
